# Reply to Letter to Editor by Elumalai et al. re : "Ginger (*Zingiber officinale* roscoe) extract could upregulate the renal expression of NRF2 and TNFα and prevents ethanol-induced toxicity in rat kidney" 

**DOI:** 10.22038/AJP.2022.66674.3146

**Published:** 2023

**Authors:** Abolfazl Akbari

**Affiliations:** Department of Physiology, School of Veterinary Medicine, Shiraz University, Shiraz, Iran

**Keywords:** Ginger, Ethanol, Kidney

**Dear editor**

Thanks for your valuable comment on the article entitled: “Ginger (*Zingiber officinale* roscoe) extract could upregulate the renal expression of NRF2 and TNFα and prevents ethanol-induced toxicity in rat kidney”. We reviewed your comment. In the beginning, I think it is necessary to first give an overview of NRF2 and its expression.

Nuclear factor erythroid 2–related factor 2 (Nrf2) is a transcription factor that regulates important antioxidant and phase II detoxification genes against oxidative stress (Tonelli et al., 2017 ▶). Nrf2 is involved in the regulation of (1) production, utilization, and regeneration of glutathione (GSH) and thioredoxin (TXN), (2) nicotinamide adenine dinucleotide phosphate (NADPH) regeneration, (3) heme and iron metabolism, (4) reactive oxygen species (ROS) and xenobiotic detoxification (Tonelli et al., 2017 ▶). Nrf2 activity is subjected to regulation at various levels including protein stability, transcription, and post-transcription (Tonelli et al., 2017 ▶; Li et al., 2019 ▶). Regulation of Nrf2 protein stability level occurs mainly by keap-1-dependent and keap-1-independent pathways. In addition to the modulation of Nrf2 protein stability, regulation of Nrf2 signaling occurs at the transcriptional level. The transcriptional factors involved include the aryl hydrocarbon receptor (AhR), NF-κB, and Nrf2 itself (Li et al., 2019 ▶). The transcription of the Nrf2 gene is found to be activated by AhR (Li et al., 2019 ▶), and the expression of AhR may be decreased by ethanol (Zhang et al., 2012 ▶). Nrf2 gene promoter also contains a binding site for NF-κB, and NF-κB subunits p50 and p65 induce transactivation of the Nrf2 gene (Rushworth et al., 2012 ▶). This explains the activation of Nrf2 by NF-κB-activating inflammatory cytokines. Although NF-κB activates Nrf2, Nrf2 activation attenuates NF-κB signaling, suggesting a cross-talk between Nrf2 and NF-κB (Cuadrado et al., 2014 ▶). Inhibition of NF-κB signaling by Nrf2 may contribute, at least partly, to the anti-inflammatory function of Nrf2 activators, such as sulforaphane (Sun et al., 2015 ▶). How Nrf2 suppresses NF-κB signaling remains unknown. It is suggested that Nrf2 activation may shift the cellular redox status to a more reducing state due to increased expression of antioxidants (Li et al., 2019 ▶), and we know that ethanol or its metabolic products may disrupt these conditions by producing different species of free radicals (Comporti et al., 2010 ▶). Nrf2 autoregulation is another mechanism that regulates the transcription of this gene. Due to the presence of ARE-like sequences in the promoter region of the Nrf2 gene, Nrf2 may activate its own gene expression, leading to increased production of Nrf2 protein (Kwak et al., 2002 ▶). This represents a positive feedback mechanism. On the other hand, Nrf2 may stimulate Keap1 gene expression for its own degradation (Lee et al., 2007 ▶). This negative feedback is a mechanism to control the undue expression of Nrf2 and uncontrolled Nrf2 signaling (Lee et al., 2007 ▶). In fact, these descriptions show that the expression of this gene can be controlled by different and interconnected cellular mechanisms that are involved in various pathophysiological events (Tebay et al., 2015 ▶; Wu et al., 2012 ▶). *In vivo* and *in vitro* studies showed that interventions such as ethanol consumption (Wu et al., 2012 ▶, Gong and Cederbaum, 2006a ▶, Dong et al., 2008 ▶), smoking (Knörr-Wittmann et al., 2005 ▶), or exposure to heavy metals (He et al., 2007 ▶; Korashy and El-Kadi, 2006 ▶) have led to different changes in the expression of this gene. The examination of each of these studies shows contradictory results regarding the expression of this gene, similar to our study. In addition, we were aware, based on studies by other researchers and previous studies on the expression of this gene that the results presented in our article may have occurred contrary to what has been expected so far. Nevertheless, we reported what we achieved. In addition, it should be noted that despite the results of all *in vivo* and *in vivo* studies, we face a complex and dynamic biological system in the face of harmful interventions that can individually produce adaptations and compensatory responses, and conflicting results that justify many unexpected data. Therefore, reporting a result in a study that is different from other studies is not far from the mind and is usually seen in many studies in different fields.

To answer to the question “why the expression of this gene is increased in the presence of ethanol? Despite the hypotheses that exist in this field”, we have not yet encountered a study that has been conducted specifically for this purpose. However, Gong et al. (2006) ▶ reported that the induction of CYP2E1 by ethanol is one pathway through which, ethanol generates oxidative stress. They also suggested that the levels of protein and mRNA Nrf2 are increased when CYP2E1 is elevated, and Nrf2 plays a key role in the adaptive response against increased oxidative stress caused by CYP2E1 (Gong and Cederbaum, 2006a ▶). Dong et al. (2008) ▶ also reported that maternal ethanol treatment increased both Nrf2 protein levels and Nrf2-ARE binding in mouse embryos. It has also resulted in a moderate increase in the mRNA expression of Nrf2 downstream target genes (Dong et al., 2008 ▶). Because exposure to ethanol results in the generation of ROS which are known to activate Nrf2 (Kensler et al., 2007 ▶), the observed Nrf2 activation was expected. This response is not unique to ethanol-exposed embryos. Similar effects have been observed in cells treated with a number of other toxic chemicals, including heavy metals (He et al., 2007 ▶; Korashy and El-Kadi, 2006 ▶), cigarette smoke (Knörr-Wittmann et al., 2005 ▶), and arachidonic acid (Gong and Cederbaum, 2006b ▶). Of particular interest to this study is that an increase in Nrf2 protein has also been observed in livers and hepatocytes of alcohol-fed mice and rats (Gong and Cederbaum, 2006a ▶).

However, another contradiction that can be seen in the results of this study is the decrease in the activity of antioxidant enzymes despite the high level of expression of the Nrf2 gene. These results can be easily described and interpreted. A decrease in the activity of antioxidant enzymes is actually due to their use to scavenge free radicals produced due to incomplete ethanol metabolism and the increase in the expression of the Nrf2s gene actually indicates a compensatory response to improve the response capacity of the antioxidant system and increase the activity of these enzymes. 

## Conflict of interest

The author declares that he has no conflicts of interest to disclose. 
